# Gleanings from Our Exchanges

**Published:** 1873-09-01

**Authors:** 


On Skin Diseases, by Dr. Hardy. — On the 13th of June, Dr. Hardy (*Mouvement Med.*, 21st June) delivered a clinical lecture on some cases occurring in his wards at the Hop. St. Louis, of Paris, which has been translated for the *Medical Press*, as follows:

"In No. 20, we have a patient who has exhibited for fifteen days past an affection which has commenced by a voluminous stain, the size of a franc, developed on the superior part of the neck. This circular stain has become larger for some days past. Beside it we see a second and third stain, both of which are concentric, and also have recently been enlarged. Upon the primitive stain, some white pustules which covered it have burst, and have left crusts. At present, the disease appears as if formed by four stains, rounded, independent of which the principal presents a crust, which results from the dessication of the vesico-pustules. With respect to symptoms in the locality of the stains, the patient speaks of pain, prickling, and itching. The general health is not altered.

"What you see there, gentlemen, is a *herpes circinatus* characterized by red, rounded stains, which tend to become larger, on which pustules or vesico-pustules have formed, which tend to burst, and forming thus crusts by the dessication of these contents.

"If the disease were to continue, you would see no longer two or three round stains, but one sole stain, formed by the re-union of the three round stains.

"Herpes circinatus is principally developed on the neck and on the hands; it is a parasitic disease, determined by the evolution in the scales of the epidermis, of the *tricophyton*, a peculiar agent of several diseases, such as sycosis, herpes tonsurane, and herpes circinatus, which constitute three degrees of one and the same malady, produced by the presence of this one and identical mushroom.

"Herpes circinatus is cured when the parasite is destroyed; the first indication is to appease the acute inflammation of the outset by means of cataplasms of starch; and only then do we employ a parasiticide; which is, at one time, a mixture of sulphur, camphor, and lard, at another, a mixture of turbith mineral, or dento-sulphate of mercury with camphor and lard; mercurial ointment is also used; but I prefer the two first. If you notice any weakness in the health, you may give tonics, bitters, etc.

"Beside this case of herpes circinatus, we have, in No. 18, the third variety, which you are already acquainted with. This patient has, on the face, swelling and induration; the hairs have fallen off; you will see and will feel local indurations, which are the result of inflammation of the hair follicles, and an œdema caused by infiltration of the cellular tissue. The hairs have the following characters: They are slightly adherent, are easily pulled out, and at one point the epidermic sheath is whitish; an excellent diagnostic sign, for the mushroom is found on this part, and it is there that the spores of the fungus may be found. With this case of sycosis we see arising in the same patient herpes circinatus, as in No. 20.

"The first part of the treatment has been performed (epilation). The second consists in parasiticide lotions, such as those of corrosive sublimate, one gramme (15 grains) in 500 grammes (15 ounces) of water. This I make to soak into the interior of the hair follicles, after first of all pulling out many of the spores along with the hairs; besides, I employ parasiticide ointment in friction, which I have mentioned in the case of herpes circinatus.

"Most generally, as it is a local disease, there is no internal treatment. The hairs grow again in from fifteen days to three weeks, and it is an error to suppose that there are permanent cavities; they always grow more luxuriantly than before. We must not forget that this disease is very tenacious, and that there remains always some spores, so that we are forced to epilate two or three times; the characters of the disease are less and less marked, and yet at length *nil.*

"In No. 35, we have a man who is the example of a disease of which I have already shown you examples; but it is only after seeing two or three cases of this disease, that we begin to recognize it well.

"This affection, which is characterized by papules covered by a little black crust, is called *prurigo*, a disease which is always secondary. Some very large papules are distributed a little over the whole body, but especially over the shoulders, where you see linear excoriations caused by the nails of the patient, on account of the atrocious itching caused, for, if I may thus express myself, that is the anatomical lesion of the itching. Thus spread abroad, prurigo is the result of the residence of a parasite, whether an acarus or louse, a diagnosis at which we arrive by an attentive examination.

"You know that the seats of predilection of the acarus are the inter-digital spaces, the anterior part of the body, etc., and that there exist characteristic furrows. Well, this patient has no eruption on the hands; and the eruption is less intense on the anterior than on the posterior surface of the body, I can, therefore, affirm that the cause of the disease is a louse. It is useless to go in search of the *corpus delicti*, this is enough; and besides, it is not always easy to find the parasite; in all cases we find it in the clothing, and not on the skin.

"This disease, which not only attacks the poorer classes, but also the richer classes of society, has, then, as diagnostic marks, great papules, linear excoriations, and itching so insupportable that patients are forced to scratch themselves; these scratchings produce a certain pleasant sensation and favor sleep.

"This patient has suffered from the disease three months, and contracted it in furnished lodgings, where the bed was occupied by an individual with the same complaint.

"For treatment, we employ fumigations, and powder his clothes and bed with powder of stavisagre.

"We must not forget the tenacity of these parasites; atmospheric conditions, the weak state of health of the patient, and sounds of any kind are causes of relapse.

"Here, gentlemen, is another parasitic affection, *itch*, in No. 27. This patient has been rubbed; but it is not of the powerful eruption that I desire to speak, but of the accessory eruption which is important for diagnosis; I would speak of the *ecthyma* on the arm, and of the *prurigo* on the back, for the acarus and pediculus often keep house together.

"The characters of this ecthyma are as follows: Rounded pustules, surrounded by a red areola, and presenting a small black central point with crusts. The ecthyma is situated generally on the buttocks and on the forearms; in our patient it is seated at the wrist, and only there; it is the pustule of variola, without any imbilication but a little black point, and the crusts are surrounded by a red circle. A pustular eruption at the wrist suffices for the diagnosis of itch.

"To combat these consecutive inflammations, we employ anti-phlogistics and baths. When there remain some acari, after friction has been used, we may require to rub two or three times."—*The Doctor.*

Abnormal Behavior of Albuminous Urine Under the Usual Tests. — Dr. Brown-Sequard (*Archives of Scientific and Practical Medicine*) points out a possible source of error in applying the usual tests for albumen in the urine. It is a well-known fact that boiling alone is not always sufficient to cause coagulation of albumen, even when the reaction of the urine is decidedly acid. In such cases, however, the subsequent addition of nitric acid, with a renewed application of heat, will generally produce a precipitate. Dr. Brown-Sequard states that in several cases that have come under his observation, he has demonstrated the presence of albumen by adding nitric acid and then applying heat, but has failed to obtain a precipitate by heat alone, or by nitric acid (and heat) after the specimen had been once boiled. There must be, therefore, a modification of albumen, which, so far from being coagulated, actually loses its coagulability by boiling.

Excision of Superior Maxillary.—John P. Wall, M.D., of Tampa, Florida (*Atlanta Med. and Surg. Jour.*), records a case of excision of the superior maxillary bone, the patient being a female child of eight years. The chief interest of the case consisted in the absence of any kind of pain, the rapidity of the growth without known cause, and the rarity of such a diseased condition of this bone in children.

A bequest of $54,575.11 was made to the Hartford Hospital, by the will of the late Chester Adams, of Hartford, Conn.

Mix the acid with three pints of water, pour this upon the powder, and set the mixture aside for a few days in a warm place; then add to it three pints of strong alcohol (95 per ct.) and macerate it for a few days longer. After this pour part of the mixture upon a muslin strainer; press the liquid out, and pack the residue of the strainer firmly into a cylindrical glass percolator, having a broad base; pour on to this foundation the rest of the mixture, together with the strained liquid, and then a mixture of equal parts of strong alcohol and water, until spirits percolate.

"This tincture conforms in the proportion of two troy ounces to one pint of the finished product, making it similar in this respect to the strength of most of the other tinctures."

### Gleanings from Our Exchanges.

A New Method of Producing Local Anæsthesia.—Dr. Horvath, of Kieff, lately proposed in the *Centralblatt fur die Medicinischen Wissenschaften*, a new method of producing local anæsthesia. If the hand be immersed for a short time in ice water, severe pain is caused. But in experiments made in reducing the temperature of frogs by means of cold alcohol, Dr. Horvarth found that no such pain was produced when the hand was immersed in cold alcohol, not even when the temperature of the alcohol was as low as 5° C. Glycerine was found to possess a similar property. Ether caused pain, and quicksilver more acute pain still, causing the speedy withdrawal of the finger when plunged into this liquid at a temperature of 3°. It was next ascertained that, when the finger was held for quite a long while in alcohol having a temperature of 5° C., no pain was experienced. Moreover, although the faintest touch was distinctly perceived in his finger, no pain was experienced from sharp pricks. This seemed to show that the application of cold alcohol has the effect of depriving the part of the special sensibility to pain, without, however, impairing the delicacy of the general tactile sensation,which, as is well known, resides in the superficial integument. This apparent possibility of the artificial separation of these two nervous functions, viz., the tactile sensation and the sensation of pain, and the temporary suspension of the latter, seemed important in a physiological point of view, and also of no small practical utility in allaying certain forms of local pain, more especially that caused by burns, and surgical operations. Dr. Horvath had an opportunity of testing the value of this application to burns on his own person, as well as upon others, and not only was all pain instantly allayed, directly the part was immersed in alcohol, but it was found that the wound very speedily began to assume a more healthy appearance, the surrounding redness rapidly failing. These observations seem of considerable importance, and we commend them to surgeons, both in reference to burns and superficial operations. —*The Doctor*.

Method of Treating Functional Dyspepsia, Anæmia and Chlorosis.— Brown-Sequard has employed successfully a method which consists in giving at one time a small quantity only of liquid or solid food, and that at regular intervals, varying from ten to twenty minutes or half an hour. The articles which he recommends for this mode of dieting are roasted or broiled meats, eggs, well cooked bread, milk, with butter and cheese, and a very small quantity of vegetables and fruits. This mode of alimentation should be continued for two or three weeks, after which the patient may be allowed to return gradually to the usual mode of three meals daily. Dr. Brown-Sequard has not always succeeded with this mode of treating the conditions above named, but in no case has it produced aggravation of symptoms. He recommends its trial in the vomiting of pregnancy, having had several cases in which it succeeded.—*Bulletin de Therapeutique.*

Use of Ozocerite.—Ozocerite, or vegetable wax, or combustible earth, is a paraffine or hydrocarbon, found in Moravia, in Valachia, in Caucasus, near to the Caspian Sea, and can be used for making illuminating gas. In its raw state this substance is of a dirty green, of slight density and fibrous structure. Kneaded in the hands for a few moments, it assumes the appearance of ordinary wax. It can be moulded, and with a wick can easily be made into candles. For therapeutical uses, either the crude ozocerite or its yellow oil is used.

This substance has upon diseases of the skin an action resembling somewhat that of pitch, without being so harsh. It can be employed mixed with glycerine or with linseed oil. Used in the form of an inunction, it is specially useful in psoriasis; also in chronic cutaneous disorders, such, for example, as eczema, tinea tonsurans, and ringworm.—*Giornal Ital. del Malat. Ven. et del Pelle*, Apr.—*Medical Record.*

SYRUP OF THE BROMIDE OF IRON.— Take of bromine three troy ounces; iron (in the form of wire) 350 grains; distilled water, three fluid ounces; citrate of potash, six troy ounces, or q. s.; syrup, a sufficient quantity. Put the iron, with the distilled water, in a flask, and add of the bromine two troy ounces, in small portions at a time, allowing the mixture to cool, if necessary, before the addition of each fresh portion. When the odor of bromine has disappeared, warm the mixture gently, until it assumes a green color; filter and add the remainder of the bromine. Transfer the resulting solution of ferric bromide to a porcelain mortar, and add gradually, with constant trituration, citrate of potash, until the color of the mixture changes from brown to green; finally, add simple syrup to make up thirty fluid ounces. The syrup thus prepared corresponds nearly in strength with the syrup of the iodide of iron, and may be administered in doses of twenty to thirty drops. It is elegant in appearance, not disagreeable in taste, and is not incompatible with alkalies or with tannic acid.

NATURE OF MUMPS. — Dr. Bouchut, in a note communicated to the Academy of Sciences by Claude Bernard, states that parotitis is simply a salivary retention due to catarrhal inflammation of the excreting canal of the parotid.

